# A mixed-flow model for heterogeneous vehicles enforcing a movement control protocol utilizing a vehicular size-based equilibrium speed function

**DOI:** 10.1016/j.heliyon.2024.e23975

**Published:** 2024-01-04

**Authors:** Md Anowar Hossain, Jun Tanimoto

**Affiliations:** a Interdisciplinary Graduate School of Engineering Sciences, Kyushu University, Kasuga-koen, Kasuga-shi, Fukuoka, 816-8580, Japan; b Faculty of Engineering Sciences, Kyushu University, Kasuga-koen, Kasuga-shi, Fukuoka, 816-8580, Japan

**Keywords:** Mixed-flow macroscopic traffic model, Diverse vehicle capability, Equilibrium speed function associated with vehicle's sizes

## Abstract

This work addressed the effect of heterogeneous vehicle sizes on traffic flow fields by introducing a movement control protocol. Considering a continuum traffic model, a new equilibrium velocity function that is dependent on traffic density was introduced to account for the effect of vehicle size. The established model showed a quantitative comparison between the Optimal Velocity and Full Velocity Difference models. A neutral stability test was carried out to evaluate the model's capability of neutralizing flow fields. The density wave behavior near a critical point was portrayed by deducing the Korteweg–de Vries–Burgers equation through a nonlinear analysis. A series of numerical simulations, the outcomes of which agreed well with the analytical results, was performed to observe the overall flow field scenario.

## Highlights

• A mixed-flow continuum traffic model introducing a new movement control protocol based on vehicle size is presented.
• A new equilibrium velocity function is introduced to account for the effects of heterogeneous vehicle sizes on flow fields.

## 1 Introduction

In the last few decades, transport authorities have attempted to ensure an appropriate balance between the demand and supply of transport facilities for citizens. However, transport facilities face numerous issues, including limited resources and poor management. Therefore, several traffic models have been developed based on theories and experiments, including microscopic models [[1], [2], [3], [4], [5], [6], [7]], macroscopic models [[8], [9], [10], [11], [12], [13]], lattice hydrodynamical models [[14], [15], [16], [17], [18]], and cellular automaton (CA) models [[19], [20], [21], [22], [23], [24]]. Most of these models have been validated under an ideal traffic flow environment with homogeneous vehicle sizes and capabilities, which could not ensure an actual traffic flow field. This type of flow field investigation may only be feasible for automatic cruising vehicles, including homogeneous vehicles, and is not a good fit for mixed-flow fields. But there are some brilliant works [[25], [26], [27], [28]] where the authors studied various traffic models in microscopic and macroscopic systems that inspired and assisted us to investigate the heterogeneous traffic flow field. There are some other outstanding studies [[29], [30], [31]] that are deeply rooted in heterogeneous traffic flow systems. C. Zhai at. El. investigated the traffic flow field in various ways [[32], [33], [34], [35]], and these works heavily boosted us to investigate the traffic flow field for heterogeneous vehicles. However, vehicles of various sizes and capabilities have been observed on inner city roads, with small vehicles, such as motorbike taxis, and auto rickshaws, traversing high-density regions. Extensive studies have explored mixed-flow traffic fields by combining CA models and human-driven vehicles. However, no investigation has focused on how traffic flow fields and vehicle movement capabilities could be affected by heterogeneous vehicle sizes (HVS). Therefore, the current work addresses such a research gap.

In 1955 beginning of the traffic flow history, Lighthill, Whitham, and Richard established a continuum traffic model known as the LWR model [8,9] relying on the 1^st^order bernoulli's law shown in Eq. (1). The following mathematical expression for the LWR model can be written:

(1) $$\frac{\partial \rho }{\partial t}+\frac{\partial \left(\rho v\right)}{\partial x}=0\text{,}$$

where x, v, ρ, and t mean the space, speed, density, and time, respectively, of a traffic flow field.

Furthermore, Payne and Zhang H. M [10,36]. reported that the LWR model fails to describe the nonequilibrium state for actual traffic flow fields and suggested a higher-order continuity equation shown in Eq. (2), which can eliminate the limitations of the LWR model. The following mathematical formulation can be written for this model:

(2) $$\frac{\partial v}{\partial t}+v\frac{\partial v}{\partial x}=-\frac{\mu }{\rho T}\frac{\partial \rho }{\partial x}+\frac{v_{e}-v}{T}\text{,}$$

$$v_{t}+\left(v+2\beta C\left(\rho \right)\right)v_{x}+\frac{C^{2}\left(\rho \right)}{\rho }\rho_{x}=\frac{V_{e}\left(\rho \right)-v}{T}+\mu \left(\rho \right)v_{xx}\text{,}$$

where T and μ are the relaxation time and anticipation parameter, respectively.

Bando et al. [1] developed a traffic model namely called the optimal velocity (OV) model focusing on individual vehicles from a Lagrangian viewpoint. The model was formulated in the simplest way by considering homogeneous vehicle capabilities, constant driver sensitivity, unique lane scheme, and prohibited passing. This is the fundamental equation for OV model:

(3) $$\frac{dv_{n}\left(t\right)}{dt}=a\left[V\left[{\Delta x}_{n}\left(t\right)\right]-v_{n}\left(t\right)\right]\text{,}$$

where *a* denotes the constant the sensitivity for the driver, $v_{n}\left(t\right)$ indicates the n th car's velocity at time t, $V\left({\Delta x}_{n}\left(t\right)\right)$ is the n th vehicle's OV, and ${\Delta x}_{n}\left(t\right)$ means the headway distance of the n th car at *t* time that is quantified by following expression: ${\Delta x}_{n}\left(t\right)=x_{n+1}\left(t\right)-x_{n}\left(t\right)$.

The OV model explained in Eq. (3) offers a new gateway for the analysis of traffic flow fields, but it has a crucial limitation in cases of acceleration and deceleration with rapid changes, which seems unrealistic. Helbing and Tilch [2] improved the model by adding a novel term called the negative velocity difference term. However, both the negative and positive speed differences significantly impact the flow field, which has been found by Jiang et al. [3] and thus revised this model. Thereafter, the FVD model explained in Eq. (4) was proposed. The mathematical equation for the FVD model, which is given below:

(4) $$\frac{dv_{n}\left(t\right)}{dt}=a\left[V\left[{\Delta x}_{n}\left(t\right)\right]-v_{n}\left(t\right)\right]+\lambda {\Delta v}_{n}\left(t\right)\text{,}$$

where ${\Delta v}_{n}\left(t\right)$ indicates the speed gap of the *n+*1st and the *n-*th car at time t that is determined by the following expression: ${\Delta v}_{n}\left(t\right)=v_{n+1}\left(t\right)-v_{n}\left(t\right)$; and λ for the constant driver's sensitivity different from a.

In densely populated cities, citizens seek alternatives to public buses or BRTA to avoid traffic jams. Hence, the demand for motorbike taxis, which are remarkably popular in cities such as Jakarta, Dhaka, and Bangkok has increased because these vehicles can easily move on congested roads. This phenomenon should be considered in traffic models that track the history of actual traffic flow fields. The OV [1] and FVD [3] models rely on homogeneous vehicles and thus ignore systems' internal noise brought by minor players vis-à-vis major ones, i.e., homogeneous “standardized” cars. In this work, we established a car-following traffic model taking into account a vehicle's movement control protocol based on vehicle sizes, in which small-sized vehicles are allowed to move in high-density regions but heavy vehicles are not. This movement control protocol is implemented by introducing a new equilibrium velocity function and all vehicles are assumed to have the same maximum velocity.

We keep the following order to organize this paper: in section II, the physical significance and mathematical expression for the HVS model have been shaped. And in section III, the flow stabilizing capability of this model has been justified utilizing linear stability theory. Moreover, we carried out a nonlinear study and numerical investigation, which is denostrated in sections IV and V, respectively. The main findings and conclusions of the HVS model are summarized in Section VI.

### 1.1 Heterogeneous vehicle size traffic model formulation

We developed a continuum traffic flow model for mixed-flow fields [3,24] called the HVS traffic model considering the impact of vehicle size. We began the model formulation on the basis of a microscopic system. The fundamental equation for this proposed model is as follows:

(5) $$\frac{dv_{n}\left(t\right)}{dt}=a\left[V_{m}\left[{\Delta x}_{n}\left(t\right)\right]-v_{n}\left(t\right)\right]+\lambda {∙\Delta v}_{n}\left(t\right)\text{,}$$

where $V_{m}\left[{\Delta x}_{n}\left(t\right)\right]$ is the OV function of the n th car, m indicates the size of the n th vehicle, and the remaining notations are the same as those in the macroscopic model.

We performed the following transformation to convert the variables from the microscopic system to a macroscopic system:

(6) $$v_{n}\left(t\right)\to v\left(x,t\right),v_{n+1}\left(t\right)\to v\left(x+\Delta ,t\right),V_{m}\left[{\Delta x}_{n}\left(t\right)\right]\to V_{m}\left(\rho \right),V_{m}^{ˊ}\left[{\Delta x}_{n}\left(t\right)\right]\to {\bar{V}}_{m}^{ˊ}\left(h\right)\to -\rho^{2}V_{m}^{ˊ}\left(\rho \right)\text{,}$$

where ρ(x,t) and v(x,t) indicate the density and speed, respectively, in a macroscopic system; and Δ means the gap between two neighbor cars. In the macroscopic system, ρ indicates the flow field's density, whereas $\Delta x_{n}$ means the headway distances of the *n-*th car in the microscopic aproach. We have a relationship between these parameters ρ and $\Delta x_{n}$. Let us consider ρ is the local density of a flow field, then the average headway of each car is defined by $\Delta x_{n}=\frac{1}{\rho }$.

**Fig. 1:**
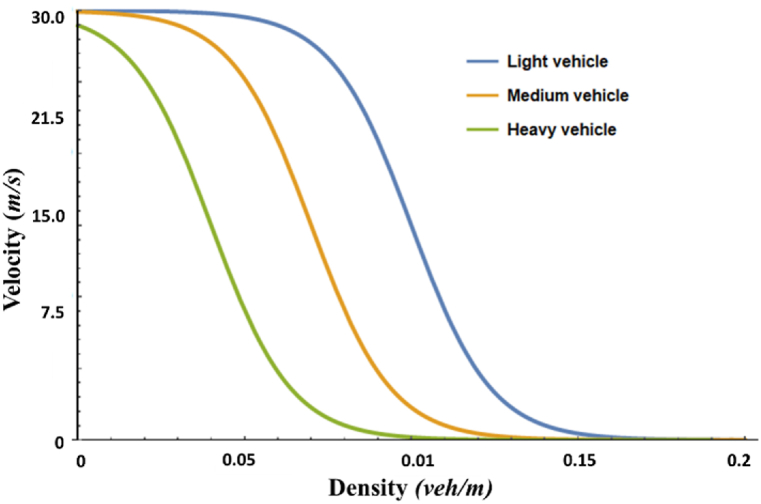
Line graphs showing the tendency of the equilibrium velocity function for three classes of vehicles, namely, light class (blue line), medium class (orange line), and heavy class (green line). (For interpretation of the references to colour in this figure legend, the reader is referred to the Web version of this article.)

To impose the heterogeneity in the traffic flow field, we focused on the vehicle's size coefficient. This is because the heavy vehicle accelerates or decelerates slowly compared to the light vehicle due to huge internal inertia. We take into account the phenomena and introduce the following equilibrium speed function enforcing movement contraol protocol based on the vehicle size, where every vehicle enjoy the same free flow speed:

(7) $$V_{k}\left(\rho \right)=v_{f}∙\left[{\left(1+\exp\frac{\left(\rho -0.02\right)/\rho_{m}-S_{k}}{0.06}\right)}^{-1}-3.72\times 10^{-6}\right]\text{,}$$

where $V_{k}\left(\rho \right)$ is the equilibrium velocity function of vehicles; $\rho_{m}$ denotes the density limit for the flow field; $v_{f}$ denotes the supreme flow speed of vehicles; and $s_{k}$ is the vehicle size coefficient. In Eq. (7), the vehicle's size coefficient does not indicate the physical length of a vehicle but the amount of the vehicle's internal inertia. In this study, we considered that $s_{k}=$ [0.05, 0.6]. The constant “0.02” is the balancing coefficient of $s_{k}$, and the remaining ones are empirical constants [37]. Fig. 1 shows the tendency of the equilibrium velocity function. For simplicity, we considered three types of vehicles in this investigation: $s_{k}=$ 0.40 for light vehicle class with equilibrium velocity denoted by $V_{L}$, $s_{k}=$ 0.25 for medium vehicle class with equilibrium velocity denoted by $V_{M}$, and $s_{k}=$ 0.10 for heavy vehicle class with equilibrium velocity denoted by $V_{H}$; herein, the vehicles have the same free flow speed $v_{f}=$ 30 m/s. The presumed function allows light vehicles to travel in high-density regions and disallows such travel for medium and heavy vehicles (Fig. 1).

The Taylor series expansion of v(x+Δ,t) can be written as follows:

(8) $$v\left(x+\Delta ,t\right)=v\left(x,t\right)+v_{x}\Delta +{\frac{1}{2}v}_{xx}\Delta^{2}\text{.}$$

Eqs. (8), (6) are substituted into Eq. (5), And then the following syplified mathematical form can be derived:

(9) $$\frac{\partial v}{\partial t}+\left(v-\lambda \Delta \right)\frac{\partial v}{\partial x}={a\left[W_{L}∙V_{L}\left(\rho \right)+W_{M}{∙V}_{M}\left(\rho \right)+W_{H}∙V_{H}\left(\rho \right)-v\right]+\frac{\lambda \Delta^{2}}{2}v}_{xx}\text{,}$$

where $W_{L}$, $W_{M}$, and $W_{H}$ are the weighted parameters for light, medium, and heavy vehicle classes, respectively, and $W_{L}+W_{M}+W_{H}=1$.

Finally, we derived this following mathematical expression from Eq. (9) and Eq. (1):

(10) $$\frac{\partial \rho }{\partial t}+\rho \frac{\partial v}{\partial x}+v\frac{\partial \rho }{\partial x}=0,\frac{\partial v}{\partial t}+\left(v-\lambda \Delta \right)\frac{\partial v}{\partial x}={a\left[W_{L}∙V_{L}\left(\rho \right)+W_{M}{∙V}_{M}\left(\rho \right)+W_{H}∙V_{H}\left(\rho \right)-v\right]+\frac{\lambda \Delta^{2}}{2}v}_{xx}\text{.}$$

This is the macroscopic form of our developed HVS traffic flow model.

### 1.2 Linear stability analysis

To investigate the flow stability, we conducted a nuetral stability analysis utizing the linear stability theory. This HVS model can be expressed in the following vector form:

(11) Ht+AHx=E,

where,

$$H=\left[\begin{array}{c} \rho \\ v \end{array}\right],A=\left[\begin{array}{cc} v & \rho \\ 0 & v-\lambda \Delta \end{array}\right]\text{,}$$

$$E=\left[\begin{array}{c} 0\\ {a\left[W_{L}∙V_{L}\left(\rho \right)+W_{M}{∙V}_{M}\left(\rho \right)+W_{H}∙V_{H}\left(\rho \right)-v\right]+\frac{\lambda \Delta^{2}}{2}v}_{xx} \end{array}\right]\text{.}$$

We obtain the following eigen values of ***A*** from Eq. (11):

(12) $$\lambda_{1}=v\;\text{and}\;\lambda_{2}=v-\lambda \Delta \text{.}$$

We assume that $\rho_{0}$ and $v_{0}$ are the initial density and velocity, respectively, for a steady flow field. Therefore, the solution for an equilibrium-state would be expressed as follows:

(13) $$\rho \left(x,t\right)=\rho_{0},v\left(x,t\right)=v_{0}\text{.}$$

By imposing a small perturbation on the uniform flow field given in Eq. (13), we obtained the following expression:

(14) $$\left(\begin{array}{c} \rho \left(x,t\right)\\ v\left(x,t\right) \end{array}\right)=\left(\begin{array}{c} \rho_{0}\\ v_{0} \end{array}\right)+\left(\begin{array}{c} {\hat{\rho }}_{k}\\ {\hat{v}}_{k} \end{array}\right)\exp\left(ikx+\sigma_{k}t\right)\text{,}$$

where k means real number and $\sigma_{k}$ indicates the wave frequency.

With the combination of Eqs. (14), (10), we have found the following equation by eliminating the higher-degree terms:

(15) $$\left(\sigma_{k}+v_{0}ik\right){\hat{\rho }}_{k}+\rho_{0}ik{\hat{v}}_{k}=0,a\left[W_{L}∙V_{L}^{ˊ}\left(\rho_{0}\right)+W_{M}∙V_{M}^{ˊ}\left(\rho_{0}\right)+W_{H}∙V_{H}^{ˊ}\left(\rho_{0}\right)\right]{\hat{\rho }}_{k}-\left[a+\frac{\lambda \Delta^{2}k^{2}}{2}+\sigma_{k}+\left(v_{0}-\lambda \Delta \right)ik\right]{\hat{v}}_{k}=0\text{.}$$

We performed a cross-product between the coefficients of ${\hat{\rho }}_{k}$ and ${\hat{v}}_{k}$. And then, we get following quadratic equation given in Eq. (16) by simplifying Eq. (15):

(16) $${\left(\sigma_{k}+v_{0}ik\right)}^{2}+\left(a+\frac{\lambda \Delta^{2}k^{2}}{2}-ik\lambda \Delta \right)\left(\sigma_{k}+v_{0}ik\right)+\left(W_{L}∙V_{L}^{ˊ}\left(\rho_{0}\right)+W_{M}∙V_{M}^{ˊ}\left(\rho_{0}\right)+W_{H}∙V_{H}^{ˊ}\left(\rho_{0}\right)\right)\left({a\rho }_{0}ik\right)=0\text{.}$$

We assume that $\sigma_{k}=\sigma_{1}\left(ik\right)+\sigma_{2}{\left(ik\right)}^{2}+\ldots$. And a negative real part has been observed for both roots of $\sigma_{k}$ in the stable flow field. These roots for the stable flow field are as follows:

(17) $$\sigma_{1}=-\left(v_{0}+W_{L}∙V_{L}^{ˊ}\left(\rho_{0}\right)+W_{M}∙V_{M}^{ˊ}\left(\rho_{0}\right)+W_{H}∙V_{H}^{ˊ}\left(\rho_{0}\right)\right).\;\sigma_{2}=\rho_{0}\left(W_{L}∙V_{L}^{ˊ}\left(\rho_{0}\right)+W_{H}∙V_{H}^{ˊ}\left(\rho_{0}\right)+W_{M}∙V_{M}^{ˊ}\left(\rho_{0}\right)\right)\text{.}$$

Therefore, the following linear stability condition can be deduced from Eq. (16):

(18) $$a_{s}=-\lambda \Delta -\rho_{0}\left(W_{L}∙V_{L}^{ˊ}\left(\rho_{0}\right)+W_{H}∙V_{H}^{ˊ}\left(\rho_{0}\right)+W_{M}∙V_{M}^{ˊ}\left(\rho_{0}\right)\right)\text{.}$$

The imaginary part of $\sigma_{k}$ can be obtain utilizing Eq. (17) is as follows:

(19) $$\text{Im}\left(\sigma_{k}\right)=-k\left(v_{0}+\rho_{0}\left(W_{L}∙V_{L}^{ˊ}\left(\rho_{0}\right)+W_{H}∙V_{H}^{ˊ}\left(\rho_{0}\right)+W_{M}∙V_{M}^{ˊ}\left(\rho_{0}\right)\right)\right)+o\left(k^{3}\right)\text{.}$$

We obtained the following critical velocity from Eq. (19), from which the disturbance moves forward:

$$c\left(\rho_{0}\right)=v_{0}+\rho_{0}\left(W_{L}∙V_{L}^{ˊ}\left(\rho_{0}\right)+W_{H}∙V_{H}^{ˊ}\left(\rho_{0}\right)+W_{M}∙V_{M}^{ˊ}\left(\rho_{0}\right)\right)\text{.}$$

**Fig. 2:**
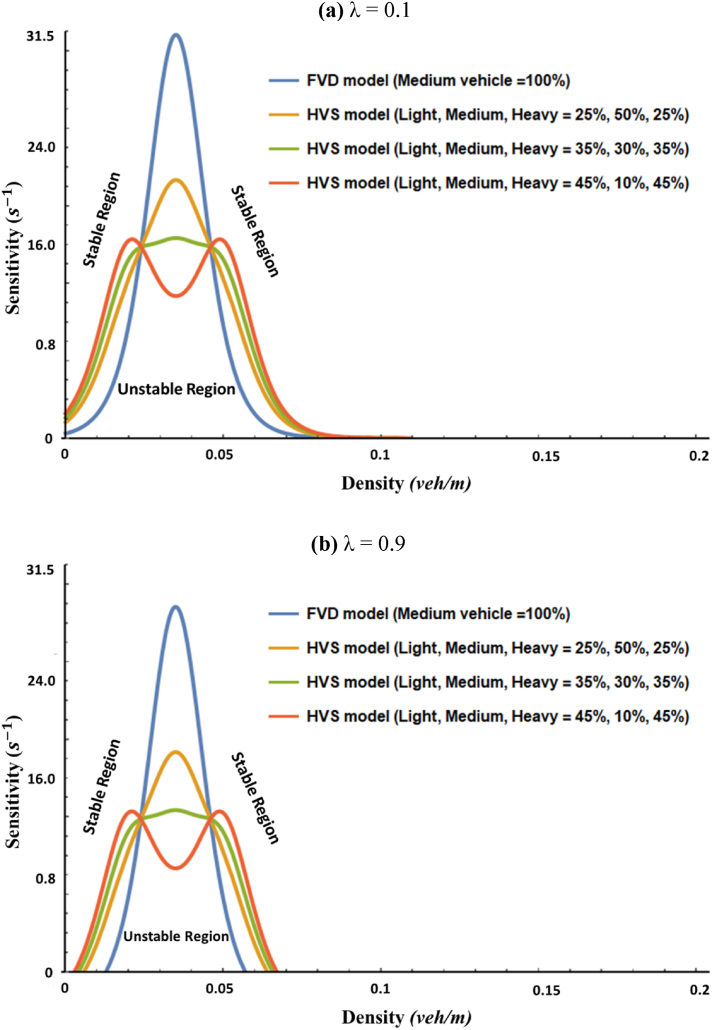
Comparison analysis of the phase diagrams of the FVD and HVS models for (a) λ=0.1 and (b) λ=0.9.

Fig. 2 dnostrated the critical line curves for neutral stability of the FVD and HVS models following the linear stability criteria explained in Eq. (18), in which we presumed panels for (a) λ=0.1 and (b) λ=0.9. At a glance, the HVS model showed higher flow stabilization capability than the fundamental FVD model, as depicted in panels (a) and (b). In both cases for figures (a) and (b), we assumed the density limit of this flow field is 0.2. And we recognized three different zones on each curve: lower density = [0.0, 0.025], intermediate density = [0.025, 0.045], and higher density = [0.045, 0.2]. The lower and higher ones had the same tendency, but the intermediate one showed a fully opposite behavior with a concave feature for the two cases, as indicated by the green and yellow lines. Notably, the flow field stabilization capability of the HVS model substantially intensified but gradually deteriorated in the lower and higher density regions as the proportions of light and heavy vehicles increased.

From a glimpse from Fig. 2 as a general tendency, our model makes a critical line with a less peak of sensitivity but with a bit wide unstable region in both lower and higher densities. It implies that the robustness toward flow-instability in an intermediate density can be significantly improved but that in both lower and higher density regions slightly gets worse.

### 1.3 Nonlinear analysis

The stability and instability activities emerged in the flow field because of a small disturbance, which is described in Eq. (18). A nonlinear study of the proposed model was conducted around neutral stability state to investigate the flow behavior. This dynamical system [38] is considered which is as follows:

(20) z=x−ct.

We obtained the following equations by replacing Eq. (20) into Eq. (10):

(21) $$-c\rho_{z}+q_{z}=0,-cv_{z}+{\left(v-\lambda \Delta \right)v}_{z}={a\left[W_{L}∙V_{L}\left(\rho \right)+W_{M}{∙V}_{M}\left(\rho \right)+W_{H}∙V_{H}\left(\rho \right)-v\right]+\frac{\lambda \Delta^{2}}{2}v}_{xx}\text{,}$$

where q=ρ.v, i.e., traffic flux is the multiple of traffic density and flow speed. The 1st and 2nd oreder derivatives of flow speed will be written as follows:

(22) $$v_{z}=\frac{c\rho_{z}}{\rho }-\frac{{q\rho }_{z}}{\rho^{2}},v_{zz}=\frac{c\rho_{zz}}{\rho }-\frac{2c\rho_{z}^{2}}{\rho^{2}}-\frac{{q\rho }_{zz}}{\rho^{2}}+\frac{{2q\rho }_{zz}^{2}}{\rho^{3}}\text{.}$$

We obtained the following equation by substituting Eq. (22) into Eq. (21):

(23) $$-c\left(\frac{c\rho_{z}}{\rho }-\frac{{q\rho }_{z}}{\rho^{2}}\right)+\left(\frac{q}{\rho }-\lambda \Delta \right)\left(\frac{c\rho_{z}}{\rho }-\frac{{q\rho }_{z}}{\rho^{2}}\right)=a\left[W_{L}∙V_{L}\left(\rho \right)+W_{M}{∙V}_{M}\left(\rho \right)+W_{H}∙V_{H}\left(\rho \right)-\frac{q}{\rho }\right]+\frac{\lambda \Delta^{2}}{2}\left(\frac{c\rho_{zz}}{\rho }-\frac{2c\rho_{z}^{2}}{\rho^{2}}-\frac{{q\rho }_{zz}}{\rho^{2}}+\frac{q\rho_{z}^{2}}{\rho^{3}}\right)\text{.}$$

We have found a flow flux q, which is as follows:

(24) $$q=\rho ∙\left[W_{L}∙V_{L}\left(\rho \right)+W_{M}{∙V}_{M}\left(\rho \right)+W_{H}∙V_{H}\left(\rho \right)\right]+b_{1}\rho_{z}+b_{2}\rho_{zz}\text{.}$$

From Eq. (23) and Eq. (24), we obtained the following:

(25) $$-c\left(\frac{c\rho_{z}}{\rho }-\frac{{\left(\rho \left[W_{L}∙V_{L}\left(\rho \right)+W_{M}{∙V}_{M}\left(\rho \right)+W_{H}∙V_{H}\left(\rho \right)\right]+b_{1}\rho_{z}+b_{2}\rho_{zz}\right)\rho }_{z}}{\rho^{2}}\right)+\left(\frac{\rho \left[W_{L}∙V_{L}\left(\rho \right)+W_{M}{∙V}_{M}\left(\rho \right)+W_{H}∙V_{H}\left(\rho \right)\right]+b_{1}\rho_{z}+b_{2}\rho_{zz}}{\rho }-\lambda \Delta \right)\left(\frac{c\rho_{z}}{\rho }-\frac{{\left(\rho \left[W_{L}∙V_{L}\left(\rho \right)+W_{M}{∙V}_{M}\left(\rho \right)+W_{H}∙V_{H}\left(\rho \right)\right]+b_{1}\rho_{z}+b_{2}\rho_{zz}\right)\rho }_{z}}{\rho^{2}}\right)=a\left[W_{L}∙V_{L}\left(\rho \right)+W_{M}{∙V}_{M}\left(\rho \right)+W_{H}∙V_{H}\left(\rho \right)-\frac{\rho \left[W_{L}∙V_{L}\left(\rho \right)+W_{M}{∙V}_{M}\left(\rho \right)+W_{H}∙V_{H}\left(\rho \right)\right]+b_{1}\rho_{z}+b_{2}\rho_{zz}}{\rho }\right]+\frac{\lambda \Delta^{2}}{2}\left(\frac{c\rho_{zz}}{\rho }-\frac{2c\rho_{z}^{2}}{\rho^{2}}-\frac{{\left(\rho \left[W_{L}∙V_{L}\left(\rho \right)+W_{M}{∙V}_{M}\left(\rho \right)+W_{H}∙V_{H}\left(\rho \right)\right]+b_{1}\rho_{z}+b_{2}\rho_{zz}\right)\rho }_{zz}}{\rho^{2}}+\frac{2\left(\rho \left[W_{L}∙V_{L}\left(\rho \right)+W_{M}{∙V}_{M}\left(\rho \right)+W_{H}∙V_{H}\left(\rho \right)\right]+b_{1}\rho_{z}+b_{2}\rho_{zz}\right)\rho_{z}^{2}}{\rho^{3}}\right)\text{.}$$

We obtained parameters $b_{1}$ and $b_{2}$ described in Eq. (26) by equating $\rho_{z}$ and $\rho_{zz}$ in Eq. (25), i.e.,

(26) $$b_{1}=\frac{c}{a}\left(c+\lambda \Delta \right)-\frac{1}{a}\left(2c+\lambda \Delta \right)\left(W_{L}∙V_{L}\left(\rho \right)+W_{M}{∙V}_{M}\left(\rho \right)+W_{H}∙V_{H}\left(\rho \right)\right)+\frac{1}{a}{\left[W_{L}∙V_{L}\left(\rho \right)+W_{M}{∙V}_{M}\left(\rho \right)+W_{H}∙V_{H}\left(\rho \right)\right]}^{2}.\;b_{2}=\frac{\lambda \Delta^{2}}{2a}\left[c-\left(W_{L}∙V_{L}\left(\rho \right)+W_{M}{∙V}_{M}\left(\rho \right)+W_{H}∙V_{H}\left(\rho \right)\right)\right]\text{.}$$

We presumed $\rho =\rho_{h}+\hat{\rho }\left(x,t\right)$ to small deviation from the neutral stability criteria. We derived the following expression applying the Taylor series and eliminating the uprer-degree terms of $\hat{\rho }$:

(27) $$\rho \left(W_{L}∙V_{L}\left(\rho \right)+W_{M}{∙V}_{M}\left(\rho \right)+W_{H}∙V_{H}\left(\rho \right)\right)\approx \rho_{h}\left(W_{L}∙V_{L}\left(\rho_{h}\right)+W_{M}{∙V}_{M}\left(\rho_{h}\right)+W_{H}∙V_{H}\left(\rho_{h}\right)\right)+{\left(\rho \left(W_{L}∙V_{L}+W_{M}{∙V}_{M}+W_{H}∙V_{H}\right)\right)}_{\rho }{│}_{\rho =\rho_{h}}\;\hat{\rho }+\frac{1}{2}{\left(\rho \left(W_{L}∙V_{L}+W_{M}{∙V}_{M}+W_{H}∙V_{H}\right)\right)}_{\rho \rho }{│}_{\rho =\rho_{h}}\;{\hat{\rho }}^{2}\text{.}$$

We obtained the following equation by replacing Eq. (27) into Eq. (24) and converting the parameter $\hat{\rho }$ into ρ:

(28) $${-c\rho }_{z}+\left[{\left(\rho \left(W_{L}∙V_{L}+W_{M}{∙V}_{M}+W_{H}∙V_{H}\right)\right)}_{\rho }+{\left(\rho \left(W_{L}∙V_{L}+W_{M}{∙V}_{M}+W_{H}∙V_{H}\right)\right)}_{\rho \rho }\rho \right]\rho_{z}+b_{1}\rho_{zz}+b_{2}\rho_{zzz}=0\text{.}$$

We performed the following transformations on Eq. (28) to obtain the standard KdV–Burgers equation form:

(29) $$U=-\left[{\left(\rho \left(W_{L}V_{L}+W_{M}{∙V}_{M}+W_{H}∙V_{H}\right)\right)}_{\rho }+{\left(\rho \left(W_{L}∙V_{L}+W_{M}{∙V}_{M}+W_{H}∙V_{H}\right)\right)}_{\rho \rho }\rho \right],X=mx,T=-mt\text{.}$$

The standard KdV–Burgers equation was obtained applying transformation given in Eq. (29):

(30) $$U_{T}+UU_{x}-mb_{1}U_{xx}-m^{2}b_{2}U_{xxx}=0\text{.}$$

where *m* indicates the constant coefficient that can be determined.

Finally, we obtained the following solution for this KdV–Burgers equation shown in Eq. (30):

$$U=-\frac{3{\left(-mb_{1}\right)}^{2}}{25\left(-m^{2}b_{2}\right)}\left[\begin{array}{c} 1+2\;\tanh\left(\pm \frac{-mb_{1}}{10m^{2}}\right)\left(X+\frac{6\left({\left(-mb_{1}\right)}^{2}\right)}{25\left(-m^{2}b_{2}\right)}T+\varepsilon_{0}\right)\\ +\tanh^{2}\left(\pm \frac{-mb_{1}}{10m^{2}}\right)\left(X+\frac{6\left({\left(-mb_{1}\right)}^{2}\right)}{25\left(-m^{2}b_{2}\right)}T+\varepsilon_{0}\right) \end{array}\right]\text{,}$$

where $\varepsilon_{0}$ denotes the random constant.

### 1.4 Numerical simulations

We performed a numerical study to confirm the theoretical investigated results and observe the history of the traffic flow field. We performed discretization on Eq. (10) to draw a definite form of the model. This is discretized form of the HVS model:

(31) $$\rho_{i}^{j+1}=\rho_{i}^{j}+\frac{\Delta t}{\Delta x}\rho_{i}^{j}\left(v_{i}^{j}-v_{i+1}^{j}\right)+\frac{\Delta t}{\Delta x}v_{i}^{j}\left(\rho_{i}^{j}-\rho_{i+1}^{j}\right)\text{.}$$

(a) If $v_{i}^{j}<c_{i}^{j}$, then

(32) $$v_{i}^{j+1}=v_{i}^{j}-\frac{\Delta t}{\Delta x}\rho_{i}^{j}\left(v_{i}^{j}-c_{i}^{j}\right)\left(v_{i+1}^{j}-v_{i}^{j}\right)+a\Delta t\left[W_{L}∙V_{L}\left(\rho_{i}^{j}\right)+W_{M}{∙V}_{M}\left(\rho_{i}^{j}\right)+W_{H}∙V_{H}\left(\rho_{i}^{j}\right)-v_{i}^{j}\right]+\frac{\Delta t\;c_{i}^{j}\Delta }{2{\left(\Delta x\right)}^{2}}\left(v_{i+1}^{j}-{2v}_{i}^{j}+v_{i-1}^{j}\right)\text{;}$$

(b) If $v_{i}^{j}\geq c_{i}^{j}$, then

(33) $$v_{i}^{j+1}=v_{i}^{j}-\frac{\Delta t}{\Delta x}\rho_{i}^{j}\left(v_{i}^{j}-c_{i}^{j}\right)\left(v_{i}^{j}-v_{i-1}^{j}\right)+a\Delta t\left[W_{L}∙V_{L}\left(\rho_{i}^{j}\right)+W_{M}{∙V}_{M}\left(\rho_{i}^{j}\right)+W_{H}∙V_{H}\left(\rho_{i}^{j}\right)-v_{i}^{j}\right]+\frac{\Delta t\;c_{i}^{j}\Delta }{2{\left(\Delta x\right)}^{2}}\left(v_{i+1}^{j}-{2v}_{i}^{j}+v_{i-1}^{j}\right)\text{,}$$

where $c_{i}^{j}=\frac{\lambda }{\rho_{i}^{j}}$ is presumed.

In the initial condition, a slight noise was imposed considering the average density $\rho_{0}$, and it led to the zig-zak phenomenon in the traffic flow field, as described in the following equation [37]:

(34) $$\rho \left(x,0\right)=\rho_{0}+\Delta \rho_{0}\left\{\cos\;h^{-2}\left[\frac{160}{L}\left(x-\frac{5L}{16}\right)\right]-\frac{1}{4}\cos\;h^{-2}\left[\frac{40}{L}\left(x-\frac{11L}{32}\right)\right]\right\}\text{.}$$

where L is the length of the domain; ${\;\rho }_{0}$ and $\Delta \rho_{0}$ denote the beginning density and density change near the equilibrium density, respectively.

We applied the following periodic boundary condition to execute out the numerical analysis:

(35) ρ(L,t)=ρ(0,t),v(L,t)=v(0,t).

We conducted this simulation following Eq. (31)− (35), and adopted the following parameter settings: L=32.2km, Δx=100m, $\rho_{m}=0.2\;\text{veh}/m$, a=1.0, Δt=1s, λ=0.5, $\Delta \rho_{0}=0.01\;\text{veh}/m$, and $v_{f}=30\;m/s$.

**Fig. 3:**
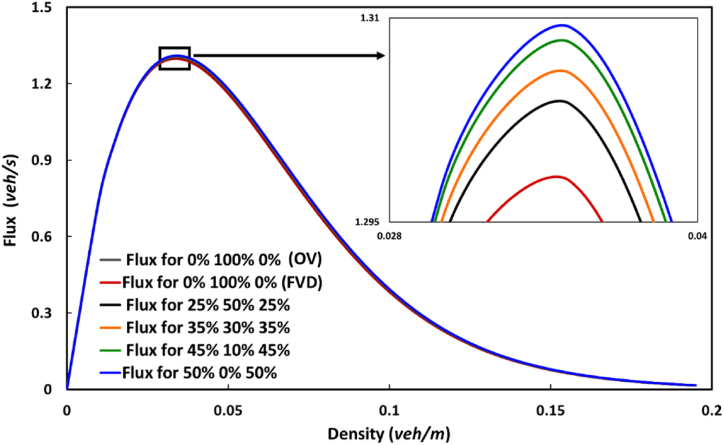
Fundamental diagrams of the traffic flux density of the OV, FVD, and HVS models with several parameter settings.

**Fig. 4:**
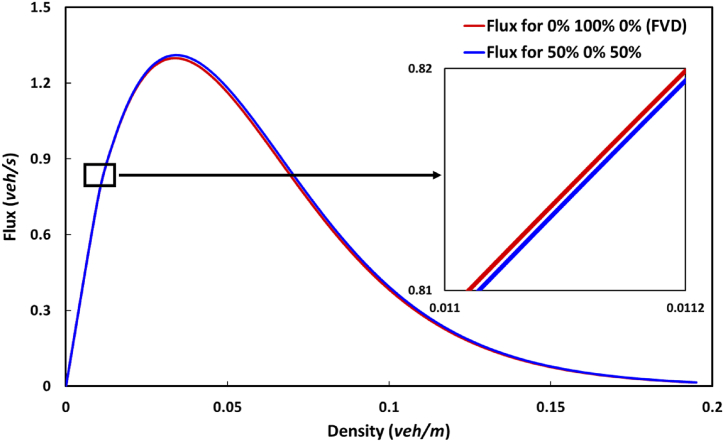
Comparison analysis of the fundamental diagrams of the traffic flux − density of the FVD and HVS models focusing on the low-density region.

Fig. 3 demonstrates the fundamental diagrams of the flow field for the traffic flux and density space at a time step of 900 s; here, the y-axis indicates the traffic flux observed in the spatial direction. A comparison analysis of these diagrams for the conventional OV and FVD traffic models and the proposed HVS model was performed with several parameter settings. The insets show a clear overview of the focal area for discussion. In this traffic flow field, the peak traffic flux shown in the insets was recorded around the density of 0.04 veh/m for all cases. In our observation, the HVS model performed better than the conventional OV and FVD [1,3] under metastable conditions. However, in the congested region, which is not shown in the inset, the HVS model was shadowed by the FVD model's flux curve. The traffic flux by the HVS model gradually improved as the proportions of light and heavy vehicles increased in the region (Fig. 3). Meanwhile, conventional traffic models were noted to be superior to the HVS model in the free flow condition, as depicted in Fig. 4. This opposite tendency was because of the introduction of an impractical high acceleration and/or deceleration characteristic by the conventional models to remove internal noise from homogeneous vehicles. However, in the synchronizing and congested regions, where synchronized traffic flow emerged automatically, the conventional models were overtaken by the HVS model, although these models performed better in the free flow phase.

**Fig. 5:**
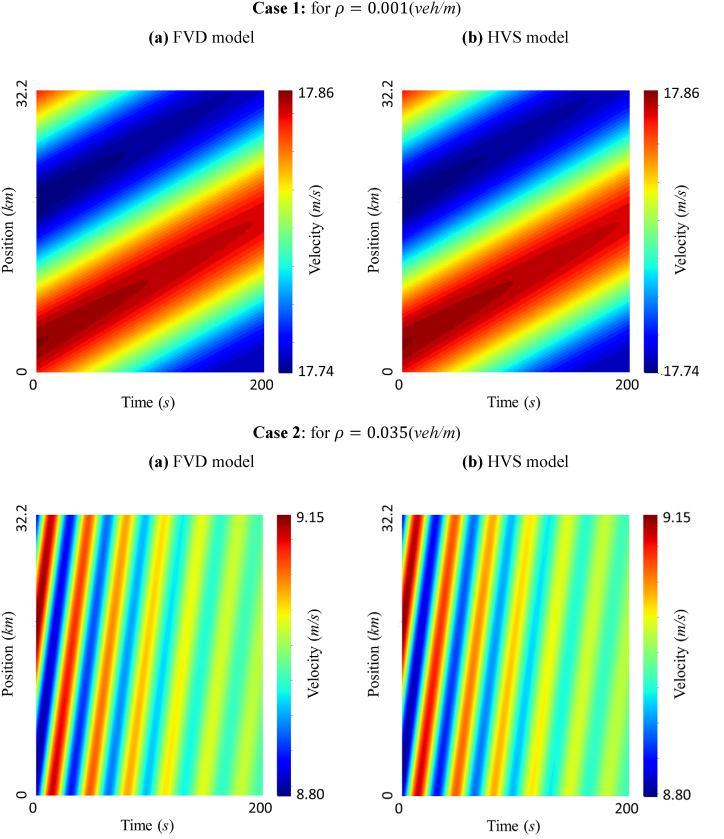
Spatiotemporal heatmap of the FVD and HVS models for (a) density =0.001 (veh/m) and (b) density =0.035*(*veh/m).

Fig. 5 shows the spatiotemporal diagram of flow velocity; case 1 for free flow state at ρ= 0.001 veh/m, and case 2 for metastable area at ρ= 0.035 veh/m. The flow field was investigated for 200 s from 800 s to 1000 s, and a comparison analysis of the FVD and HVS models was conducted. The velocity wave tendencies and amplitudes of the models were identical, but a higher average velocity was recorded for the FVD model than that for the HVS model in the free flow condition, which is depicted in panels Fig. 5 (a, b) of case 1. Nevertheless, the HVS model, relative to the FVD model, showed outstanding performance in the high-density region for metastable and congested areas, as demonstrated in panels Fig. 5 (a, b) of case 2.

**Fig. 6:**
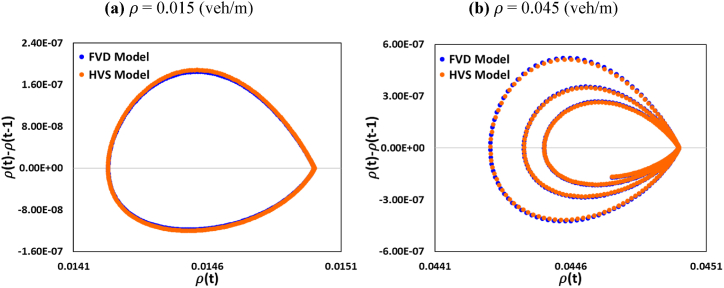
Comparison of trajectory diagram of FVD and HVS models for (a) density =0.015 (veh/m) and (b) density =0.045 (veh/m).

Fig. 6 (a, b) represents the trajectory loop diagrams of the density in the free flow condition at ρ= 0.001 veh/m and in the metastable area at ρ= 0.035 veh/m. For the synchronizing and Jamming regions, the HVS model showed a more stabilized flow field than the FVD model. Meanwhile, the conventional FVD model dominated in the free flow situations. These phenomena in Fig. 5, Fig. 6 showed complete agreement with the fundamental diagram depicted in Fig. 3, Fig. 4.

## 2 Conclusion

We developed a macroscopic traffic model called the HVS model and introduced the effect of heterogeneous vehicles size. We imposed a new movement control protocol in which the mobility capability of vehicles in high-density areas gently increases with reduced vehicle sizes. For this protocol, we introduced a new equilibrium speed function taking into account vehicle size coefficient given the same maximum velocity of vehicles. The linear stability analysis showed that the flow neutralizing proficiency of the HVS model varied with density relative to the fundamental FVD model. The developed HVS model outperformed the conventional OV and FVD models in high-density areas such as metastable and congested regions, although its superiority was different in the free flow retitiry. The flow behavior around the critical place was assessed by the nonlinear analysis, and the adopted KdV–Burgers equation suggested a wavy traffic flow pattern. Furthermore, a numerical analysis was performed to confirm the theoretical results, which showed a great agreement with the analytical solutions. This results exhibited that the proposed HVS model is highly efficient for synchronizing and jamming regions. Let us mention the limitation of the present modeling. In this study, we modeled a heterogeneous vehicle's effect on the traffic flow system depending on the vehicle's size. One obvious limitation in the present approach is a coarse classification of vehicle size; only three classes. To exactly model the real world, we do need to define the probability density function (PDF) of vehicle size to account for the model.

## Data availability

There is no associated data of this work. This study has been conducted relies on the simulation.

## CRediT authorship contribution statement

**Md Anowar Hossain:** Conceptualization, Formal analysis, Methodology, Validation, Writing – original draft. **Jun Tanimoto:** Conceptualization, Funding acquisition, Investigation, Supervision, Writing – review & editing.

## Declaration of competing interest

The authors declare that they have no known competing financial interests or personal relationships that could have appeared to influence the work reported in this paper.
